# Human mesenchymal stem cells are resistant to cytotoxic and genotoxic effects of cisplatin *in vitro*

**DOI:** 10.1590/1678-4685-GMB-2015-0057

**Published:** 2016

**Authors:** Bruno Corrêa Bellagamba, Bianca Regina Ribas de Abreu, Ivana Grivicich, Carolina Franke Markarian, Eduardo Chem, Melissa Camassola, Nance Beyer Nardi, Rafael Rodrigues Dihl

**Affiliations:** 1 Programa de Pós-Graduação em Genética e Biologia Molecular, Universidade Federal do Rio Grande do Sul, Porto Alegre, RS, Brazil; 2 Programa de Pós-Graduação em Biologia Celular e Molecular Aplicada à Saúde, Universidade Luterana do Brasil, Canoas, RS, Brazil; 3 Complexo Hospitalar Santa Casa de Porto Alegre, Porto Alegre, RS, Brazil

**Keywords:** Mesenchymal stem cells, Cisplatin, MTT, Comet assay, OVCAR-3 cells

## Abstract

Mesenchymal stem cells (MSCs) are known for their important properties involving multilineage differentiation potential., trophic factor secretion and localization along various organs and tissues. On the dark side, MSCs play a distinguished role in tumor microenvironments by differentiating into tumor-associated fibroblasts or supporting tumor growth via distinct mechanisms. Cisplatin (CIS) is a drug widely applied in the treatment of a large number of cancers and is known for its cytotoxic and genotoxic effects, both *in vitro* and *in vivo*. Here we assessed the effects of CIS on MSCs and the ovarian cancer cell line OVCAR-3, by MTT and comet assays. Our results demonstrated the resistance of MSCs to cell death and DNA damage induction by CIS, which was not observed when OVCAR-3 cells were exposed to this drug.

Cisplatin, *Cis*-diamminedichloroplatinum-II, (CIS) is a widely used chemotherapeutic agent as the first line treatment against head and neck, testicular, lung, ovarian and others types of cancer (Jamieson and Lippard, 1999; Gomez-Ruiz *et al.*, 2012). Its main mechanism of action involves formation of adducts covalently linked to DNA (Sancho-Martinez*et al.*, 2012). These adducts are considered the major contributing factor to the cytotoxic effects of the drug, since they block DNA replication and transcription and, ultimately, cell division (Dasari and Tchounwou, 2014). Despite the well-established application of CIS in clinical treatments, intrinsic or acquired cell resistance to this drug is a serious problem that appears concomitant with CIS utilization (Galluzzi *et al.*, 2012).

Mesenchymal stem cells (MSCs) are post-natal stem cells found in almost all tissues in the organism (da Silva Meirelles *et al.*, 2006), including human adipose tissue (Zuk*et al.*, 2002), since they inhabit a perivascular niche (da Silva Meirelles *et al.*, 2008). MSCs can secrete trophic factors such as anti-apoptotic, immunomodulatory, angiogenic and chemo attractive molecules, which act in lesion and surrounding sites *in vivo* to promote tissue repair (Doorn *et al.*, 2012). Another property of MSCs is the potential to differentiate into bone, cartilage and adipocytes (Zhu *et al.*, 2012) according to the culture conditions. Due to their capacity to differentiate into various cell types and their paracrine effects, MSCs have emerged as a promising alternative for cell therapy and tissue engineering (Schaffler and Buchler, 2007).

While MSCs exert important roles in the maintenance of organismic homeostasis, they are also known for composing the tumor stroma and for their tropism to various types of cancer (Kucerova and Skolekova, 2013). *In vitro* analyses suggest that MSCs can stimulate tumor progression by modulating cytokine secretion, supressing the immune system, migrating to the tumor site and promoting tumor growth through paracrine factors, or by differentiating into tumor-associated fibroblasts. When MSCs are injected at the tumor site *in vivo*, they stimulate tumor growth and support metastasis, or inhibit tumorigenesis by antitumor effects involving downregulation of Akt, beta-catenin, Bcl-2, c-Myc, proliferating cell nuclear antigen and surviving, leading to reduced proliferation, G1 arrest, suppression of oncogenes and increased apoptosis (Klopp *et al.*, 2011).

Given the complexity of the tumor microenvironment and the increasing evidence for the contribution of tumor-associated fibroblasts to cancer maintenance and chemoresistance (Houthuijzen *et al.*, 2012), tumor-associated fibroblasts have been considered as promising targets for novel chemotherapeutic strategies (Samples *et al.*, 2013). Several studies have shown the cytotoxic (Smith *et al.*, 2005) and genotoxic (Unger *et al.*, 2009) effects of CIS on ovarian carcinoma cells (OCCs) and normal proliferating and non-proliferating cells (Sancho-Martinez *et al.*, 2012). However, the genotoxic effects of CIS on MSCs are still not clear, even though these cells are known for being resistant to several chemotherapeutic agents*in vitro*, including CIS (Li*et* al., 2004; Liang*et al.*, 2011). Thus, due to the well known importance of MSCs for generating tumor-associated fibroblasts and their role in the cancer microenvironment and chemoresistance, the present study aimed at evaluating the cytotoxic effect and DNA damage induction potential of CIS on human adipose-derived MSCs and OCCs line OVCAR-3 during *in vitro* cultivation.

Human adipose-derived MSCs were obtained from adipose tissue of four patients undergoing elective liposuction surgery. All patients signed an informed consent form, and the study was approved by the Research Ethics Committee of Complexo Hospitalar Santa Casa de Misericórdia de Porto Alegre. The stromal vascular fraction was isolated as described byZuk *et al.* (2002). Briefly, the liposuction material was extensively washed with phosphate buffered saline (PBS) and incubated with type I collagenase. Mono-nuclear cells resulting from tissue digestion and centrifugation were resuspended in Dulbecco's modified Eagle medium (DMEM) supplemented with HEPES (free acid, 3.7 g/L), 10% fetal bovine serum (FBS, Cultilab, São Paulo, Brazil) and 1% penicillin/streptomycin (Cell Culture Medium 1 – CCM1). Cells were seeded at 3 × 10^4^ cells/cm^2^ into tissue culture flasks and expanded at 37 °C in a humidified culture chamber with a 5% CO_2_ atmosphere, changing the culture medium every 2–3 days. Cells between passages 6 and 9 were used in all experiments. Immunophenotyping of MSCs was done using a BD FACSCalibur flow cytometer to determinate the presence/absence of the following cell markers: CD13, CD69, CD73, CD90, CD117 and HLA-DR (Figure 1). All reagents used here were from Sigma Chemical Co. (St Louis, MO, USA), unless otherwise stated. Plasticware was from TPP (Trasadingen, Switzerland).

**Figure 1:**
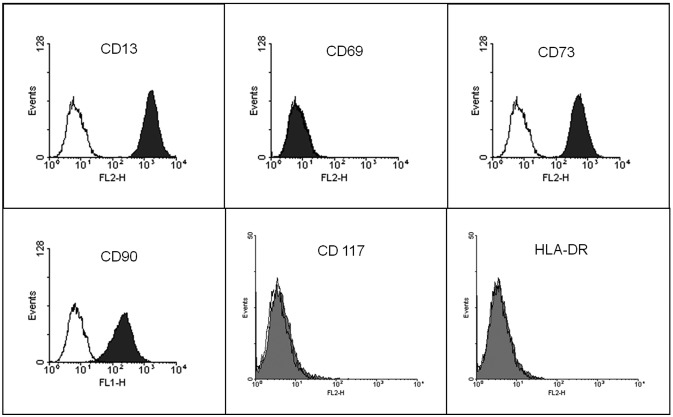
Immunophenotypic profile of cultured human adipose-derived MSCs. Cells expressed CD13, CD73 and CD90, but did not express CD69, CD117 and HLA-DR markers.

The ovarian cancer cell line OVCAR-3 was purchased from ATCC (American Type Culture Collection, Manassas, Virginia, USA) and cultured with DMEM supplemented with 10% of FBS and 1% penicillin/streptomycin (Cell Culture Medium 2 – CCM2) at 37 °C in a humidified culture chamber with 5% CO_2_. Cells were expanded according to the experiments requirement.

Cisplatin (CIS, CAS No.15663-27-1) was obtained as the clinical preparation Platistine® (Pfizer Ltda., São Paulo, Brazil). Ethyl methanesulfonate (EMS, CAS No.62-50-0) was purchased from Sigma-Aldrich. Solutions of CIS and EMS were made with CCMs immediately before use.

For the MTT assay evaluation of CIS cytotoxicity, cells were seeded in 96-well plates at a density of 3 × 10^3^ cells/well for MSCs and 5 × 10^4^ cells/well for OVCAR-3 cells and treated the following day with CIS at 0.5, 1,3,5, 10 and 50 μM dosages. CCMs, 1 and 2, were used as negative control (NC). After 72 hours, CCMs were removed and 20 μL of 3-(4,5-dimethylthiazol-2-yl)-2,5-diphenyl tetrazolium bromide (MTT) solution (5 mg/mL) was added to each well and incubated at 37°C for 2 h. Formazan crystals resulting from the cleavage of MTT were dissolved in 100 μL DMSO for 5 min with shaking. Each plate was read immediately in a microplate reader (Thermo Scientific, Waltham, MA) at a wavelength of 540 nm. Three independent experiments were performed in triplicate for each type of cell culture. Cell viability is expressed in Figure 2 as percentage of the viability of untreated cells. Since the percentage of viable cells was calculated considering the average absorbance from cells of NCs, standard deviations of these groups of treatment are variable. The determination of the 50% inhibition concentration (IC50) of CIS for each cell type was carried out by the sigmoidal fitting method (Sebaugh, 2011).

**Figure 2:**
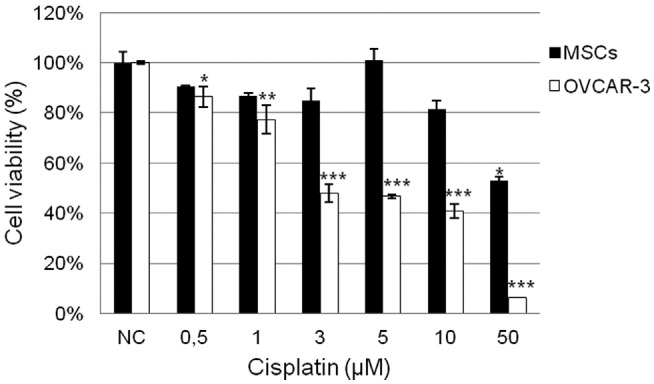
Percentage of viable cells, evaluated by MTT assay, after 72 h of exposure to increasing concentrations of cisplatin (CIS). Black bars represent MSCs and white bars correspond to OVCAR-3 cells. NC: Negative Control. *P <0.05; **P <0.01; ***P <0.001.

To detect DNA strand breaks and alkali labile as well as incomplete excision repair sites, we used the alkaline single-cell microgel electrophoresis (Comet) assay as described previously (Tice *et al.,*2000). MSCs and OVCAR-3 cells were treated for 1 and 24 h with three concentrations of CIS, 3, 5 and 10 μM. These drug dosages were chosen considering viability of at least 70% of cells treated for 24h with CIS (data not shown). We used ethyl methanesulfonate (EMS) 5 mM as positive control (PC). After treatments, viable cells were trypsinized, resuspended in 0.5% low melting agarose (Invitrogen Co, Carlsbad, CA) and distributed onto slides (Knittel Glaser, Braunschweig, Germany) previously coated with 1.5% normal melting agarose (Invitrogen Co, Carlsbad, CA). After cell lysis for 24 h in alkaline lysis buffer (10% DMSO, 1% Triton-X, 2.5 M NaCl, 10 mM Tris, 100 mM EDTA, pH 10), slides were placed in a horizontal gel electrophoresis chamber and covered with alkaline buffer (5 mM NaOH and 200 mM EDTA) at pH >13. After a 20 min period for DNA denaturation, electrophoresis was performed under standard conditions (1 V/cm, 300 mA, distance between electrodes 36 cm) for 20 min. Following neutralization at pH 7.5 (0.4 M Tris), cells were stored until analysis. All preparation steps were performed under red or yellow light to avoid DNA damage by UV light.

The slides were analyzed in an Olympus System Microscope (Model BX41) equipped with a Olympus Reflected Fluorescence System (Model U-RFL-T) and Olympus U-TV0.35XC-2 Camera (Tokyo, Japan). After coding and blinding of the slides, they were stained with ethidium bromide solution and the comets were determined by an image analysis system (Comet Assay IV, Perceptive Instruments, UK). Four slides with 25 cells (total of 100 cells) for every test sample were counted and analyzed for the Tail Length (TL) parameter to quantify the induced DNA damage. Data from TL are given in Table 1. Results are given as mean ± standard deviation (SD).

**Table 1:** Results of the Comet assay on MSCs and OVCAR-3 cells after treatment with CIS.

	MSCs	1 h treatment	24 h treatment
	Drug Concentrations	Mean ± Standard Deviation	Mean ± Standard Deviation
TL	NC	35.43 ± 5.94	35.51 ± 8.66
	CIS 3 μM	35.32 ± 8.49	23.59 **±** 9.88
	CIS 5 μM	40.05 ± 15.58	23.05 ± 7.90
	CIS 10 μM	39.11 **±** 7.86	22.17 ± 7.93
	PC	75.92 ± 48.39**	158.45 ± 22.13***
	OVCAR-3	1 h treatment	24 h treatment
	Drug Concentrations	Mean ± Standard Deviation	Mean ± Standard Deviation
TL	NC	33.21 ± 7.47	33.15 ± 8.04
	CIS 3 μM	66.62 ± 23.22**	56.05 ± 23.36*
	CIS 5 μM	68.39 ± 24.85**	72.17 ± 28.36**
	CIS 10 μM	57.39 ± 17.13*	71.18 ± 21.32**
	PC	103.86 ± 29.92***	147.30 ± 77.33***

Statistical analysis of the obtained data was performed using the SPSS software, version 13.0. To analyze differences, One Way ANOVA with Dunnett post hoc test was applied, where the drug treatments were compared against the negative control. Differences were considered statistically significant when the P-value was less than 0.05.

After 72 h of treatment, MSCs, but not OVCAR-3 cells, showed resistance to the increasing concentrations of CIS when evaluated by MTT assay (Figure 2, black bars). We assumed a reduction above 50% of cell viability for treated cells in relation to NC as indication of cytotoxicity. The cell percentage for OVCAR-3 cells was highly reduced, in comparison with the NC, showing their sensitivity to the drug (Figure 2, white bars). In addition, due to the high cytotoxic effect of CIS on OVCAR-3 cells, we calculated the IC50 value, that was found to be 2.86 μM.

We assessed genotoxic potential of CIS by Comet assay during two times of exposure (1 and 24 h) by tail length (TL) analysis, as migration distance of DNA in μm. After the two times of exposure to the drug, no DNA damage was observed in MSCs for all tested dosages, with exception of PC (Table 1). However, CIS was able to induce DNA damage in OVCAR-3 cells during the two times of exposure (Table 1). After 1 h of treatment, all dosages of CIS caused a significant increase in DNA migration (TL) in OVCAR-3 cells in comparison to NC. TL was also significantly higher in OVCAR-3 cells treated with CIS after 24 h of exposure, at 3, 5 and 10 μM dosages, in relation to NC.

In this study we demonstrated the resistance of human adipose-derived MSCs to the exposure of increasing concentrations of CIS during 72 h of *in vitro*cultivation (Figure 2, black bars). Our data are in agreement with results obtained by Liang *et al.* (2011), which showed the resistance and recovery of human adipose-derived MSCs to CIS exposure. They also demonstrated that MSCs retain their phenotypical characteristics, such as a fibroblast-like morphology and stem cell marker expression, as well as their multilineage differentiation capacity.

In contrast, CIS was able to dramatically reduce the viability of OVCAR-3 cells after 72 h of treatment (Figure 2, white bars). The IC50 value of CIS for OVCAR-3 cells was 2.86 μM, which means that CIS was, at least, 17 fold more cytotoxic for OVCAR-3 cells than for MSCs, since the highest tested dosage of CIS (50 μM) was not able to reduce MSC viability above 50%. The sensitivity of OVCAR-3 cells to CIS is in accordance with previously data demonstrated by several studies (Smith *et al.,* 2005; Karaca *et al.,* 2013).

After confirming the resistance of MSCs to CIS we chose three dosages (3, 5 and 10 μM) to evaluate the genotoxic potential of this drug on MSCs and OVCAR-3 cells. Using the Comet assay, we were able to demonstrate, for the first time, the absence of DNA damage caused by CIS on MSCs (Table 1) after 1 and 24 h of treatment, in our experimental conditions. In contrast, CIS significantly increased DNA migration of OVCAR-3 cells comets (Table 1), showing its genotoxic effect. In addition, our results demonstrate that the concentrations of CIS used in this study were not associated with retarded DNA migration, as expected by interstrand DNA cross-links, observed for treatments with concentrations above 50 μM of CIS (Almeida *et al.,* 2006; Pang *et al.,* 2007).

CIS is a strong genotoxic and mutagenic agent (Roos and Kaina, 2013). It is able to induce DNA damage in a broad range of eukaryotic cells, from *Drosophila melanogaster* to humans, either *in vitro* and *in vivo.* Because of its capacity to cause DNA adducts, CIS induced DNA strand breaks in *D. melanogaster* somatic cells*in vivo,* evaluated by the Comet assay (Garcia Sar *et al.,* 2012) and SMART test (Danesi *et al.,* 2010). A large panel of mammalian cells have already been exposed to CIS, and its genotoxic potential has been confirmed on cells from hamster (Brozovic *et al.,* 2009), mice (Narayana, 2012), rats (Mendonça *et al.,* 2010) and human normal and cancer cells (Blasiak *et al.,* 2000; Shimabukuro *et al.,* 2011). Although CIS is a widely used drug for the treatment of a broad range of cancers, tumor resistance to CIS is an issue to be surpassed. The main mechanisms of cell resistance to CIS are described as: decreasing intracellular accumulation of CIS, increasing intracellular trapping of CIS, increased repair of DNA damage or increased tolerance of DNA damage and, finally, the mixture of a variety of others and before mentioned mechanisms (Borst *et al.,*2008).

Here we demonstrated that human MSCs are strongly resistant to CIS exposure, considering this drug as a cytotoxic and genotoxic agent, but the mechanisms underlying this property are still poorly understood. It was shown that human bone marrow MSCs can be isolated from patients after high-dose or standard chemotherapy and the cells retained their MSCs characteristics (Mueller *et al.,* 2006). These authors also demonstrated that MSCs have an elevated threshold for CIS-induced apoptosis, which was characterized by a lack of caspase-9 activity in apoptotic cells and an increased p53 expression, independent of apoptosis induction (Mueller *et al.*, 2006). p73 also seems to play a role in MSC resistance to CIS, since the over-expression induction of this apoptosis regulator sensibilizes human bone marrow MSCs to CIS treatment (Liang *et al.*, 2010). Prendergast *et al.*(2011) showed that CIS can activate DNA damage response pathways, including induction of p53 and p21, and activation of PI3 kinase-related protein kinase (PIKK)-dependent phosphorylation of histone H2AX on serine 139, and replication protein A2 on serine4/serine8, in human bone marrow MSCs. Taken together, these findings indicate that the resistance of MSCs to CIS results from complex cellular pathways, involving alteration of apoptosis regulation and activation of molecules engaged in DNA repair process. Our data suggest that the tolerance of MSCs to DNA damage, potentially induced by CIS, could also be related to the resistance of these cells to the drug. Finally, we emphasize the need for further investigations aiming to elucidate the mechanisms responsible for MSC resistance to drugs, since the importance of these cells in the tumor microenvironment context is well known.
